# BloodChIP Xtra: an expanded database of comparative genome-wide transcription factor binding and gene-expression profiles in healthy human stem/progenitor subsets and leukemic cells

**DOI:** 10.1093/nar/gkad918

**Published:** 2023-10-23

**Authors:** Julie A I Thoms, Forrest C Koch, Alireza Raei, Shruthi Subramanian, Jason W H Wong, Fatemeh Vafaee, John E Pimanda

**Affiliations:** School of Biomedical Sciences, University of New South Wales, Sydney, Australia; School of Biotechnology and Biomolecular Sciences, University of New South Wales, Sydney, Australia; School of Biotechnology and Biomolecular Sciences, University of New South Wales, Sydney, Australia; School of Clinical Medicine, University of New South Wales, Sydney, Australia; School of Biomedical Sciences, Li Ka Shing Faculty of Medicine, The University of Hong Kong, Hong Kong SAR, China; School of Biotechnology and Biomolecular Sciences, University of New South Wales, Sydney, Australia; UNSW Data Science Hub, University of New South Wales, Sydney, Australia; School of Biomedical Sciences, University of New South Wales, Sydney, Australia; School of Clinical Medicine, University of New South Wales, Sydney, Australia; Haematology Department, Prince of Wales Hospital, Sydney, Australia

## Abstract

The BloodChIP Xtra database (http://bloodchipXtra.vafaeelab.com/) facilitates genome-wide exploration and visualization of transcription factor (TF) occupancy and chromatin configuration in rare primary human hematopoietic stem (HSC-MPP) and progenitor (CMP, GMP, MEP) cells and acute myeloid leukemia (AML) cell lines (KG-1, ME-1, Kasumi1, TSU-1621-MT), along with chromatin accessibility and gene expression data from these and primary patient AMLs. BloodChIP Xtra features significantly more datasets than our earlier database BloodChIP (two primary cell types and two cell lines). Improved methodologies for determining TF occupancy and chromatin accessibility have led to increased availability of data for rare primary cell types across the spectrum of healthy and AML hematopoiesis. However, there is a continuing need for these data to be integrated in an easily accessible manner for gene-based queries and use in downstream applications. Here, we provide a user-friendly database based around genome-wide binding profiles of key hematopoietic TFs and histone marks in healthy stem/progenitor cell types. These are compared with binding profiles and chromatin accessibility derived from primary and cell line AML and integrated with expression data from corresponding cell types. All queries can be exported to construct TF–gene and protein–protein networks and evaluate the association of genes with specific cellular processes.

## Graphical Abstract

**Graphical Abstract:**
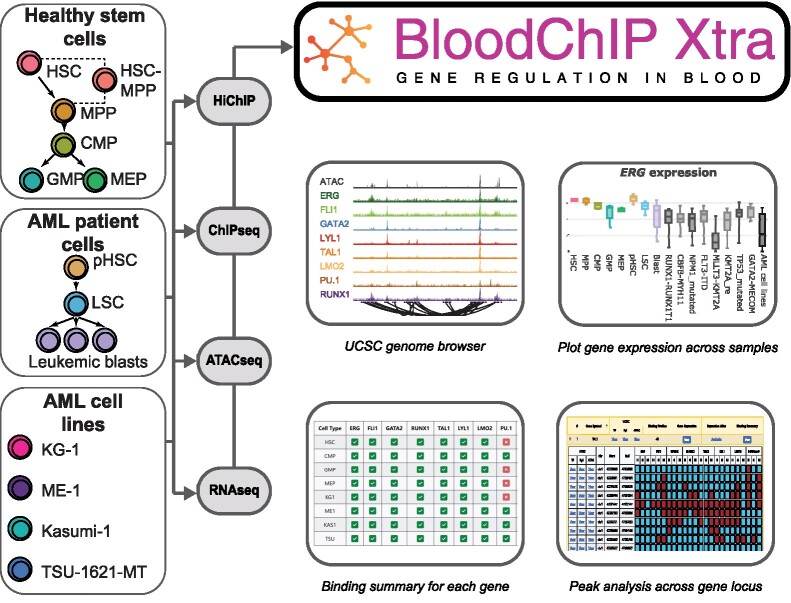

## Introduction

Transcription factors (TFs) and the *cis*-regulatory DNA sequences they bind are components of gene regulatory networks (GRNs) that control gene expression and ultimately cell identity (1–3). GRNs are comprised of genes, their associated regulatory elements (promoters and *cis*-regulatory elements (CREs) such as enhancers), and DNA binding proteins, including TFs, often as part of multi-factor regulatory complexes which are increasingly understood to contain RNA as well as proteins. The accessibility of regulatory elements is modulated by direct DNA modifications such as CpG methylation and post-translational modification of histone proteins, and even when CREs are in an open conformation the DNA sequence of such elements at least partially determines which TFs can bind (4,5). Interactions between promoters and their CREs, mediated by chromatin loops and complexes of transcriptional regulators, modulate transcriptional burst frequency and output and therefore cell identity.

Stem cells have the capacity to either self-renew or give rise to daughter cells with increasingly specialized function and restricted proliferative capacity, and changes in cell identity occurring over differentiation trajectories are directly related to altered transcriptional programs controlled by lineage specific GRNs. Hematopoiesis is one of the best characterized developmental systems in vertebrates, serving as a model system for the multi-lineage differentiation of adult stem cells. Hematopoietic stem cells (HSCs) maintain production of circulating blood cells via their capacity to either self-renew or differentiate to mature cell types. The most primitive HSCs have multi-lineage potential but give rise to progenitor cells with increasing lineage restriction (3,6). However, the components and hierarchy of the GRNs which control the identity of human adult HSCs and their progeny, and the mechanisms by which these are hijacked or reactivated in leukemic cells, remain incompletely understood.

TFs act in the context of multi-protein complexes that bind distal regulatory elements and interact with promoters to initiate, modulate, or suppress gene expression in conjunction with a plethora of chromatin modifiers and transcription initiation/elongation factors. A core set of seven TFs (heptad: ERG, FLI1, GATA2, RUNX1, TAL1, LYL1 and LMO2), all known regulators of healthy and leukemic hematopoiesis (7–14), work in combination to regulate gene expression in hematopoietic stem and progenitor cells (HSPCs). Heptad TFs also contribute to stem cell-like gene expression signatures in leukemic cells, where their presence correlates with poor patient survival (15). Knowledge of how these TFs cooperate and interact with other lineage-specific TFs as HSCs make lineage commitment decisions is vital to improving our understanding of both healthy and leukemic blood development.

To this end, we have recently generated high-quality ChIPmentation and ChIPseq data for heptad TFs, CTCF, STAG2, the master myeloid regulator PU.1, H3K27ac, H3K4me3, H3K27me3 and also H3K27ac HiChIP in rare hematopoietic subpopulations (stem/multipotent progenitors [HSC-MPP] plus common myeloid [CMP], granulocyte macrophage [GMP] and megakaryocyte erythrocyte [MEP] progenitors) spanning HSC commitment along the myeloid/erythroid lineages (5). To optimize the utility of these data, BloodChIP Xtra integrates our chromatin occupancy data with published chromatin occupancy and accessibility, plus expression data, from healthy HSPCs, primary AML and AML cell lines. These datasets significantly increase the volume of data that can be queried compared to our earlier database BloodChIP (16). All analyzed and filtered data sets can be exported for downstream analysis, and we also provide links to external resources such as the UCSC genome browser to visualize data and the Gene Expression Atlas to query expression in additional cell types.

## Material and methods

### Data sources and analysis strategy

**Figure 1.**
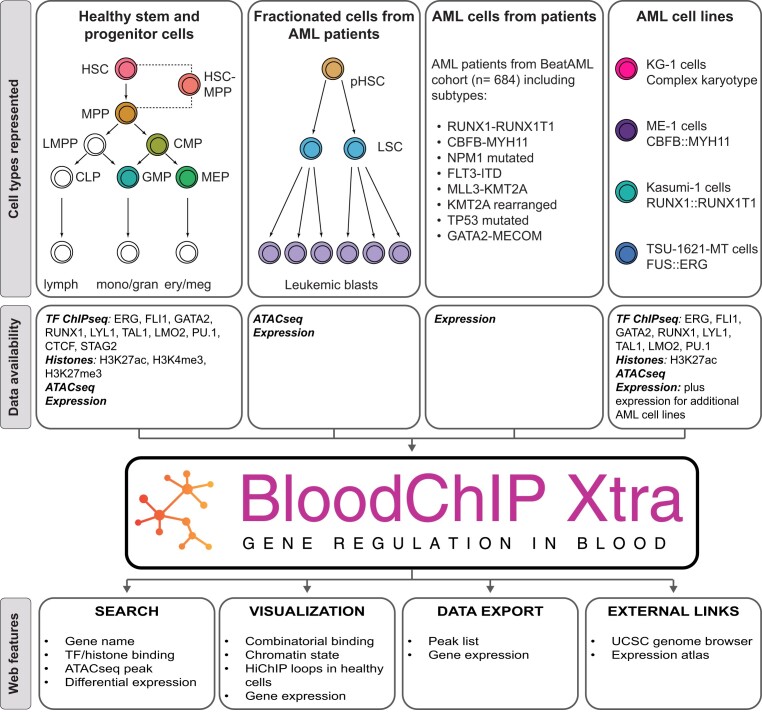
Schematic showing the datasets held by the BloodChIP Xtra database and features of the web interface. The database integrates TF ChIPseq, Histone ChIPseq, ATACseq, H3K27ac HiChIP and RNAseq expression data, while the web interface provides methods to query and visualize data. Cell types shown in white have expression data only.

**Figure 2.**
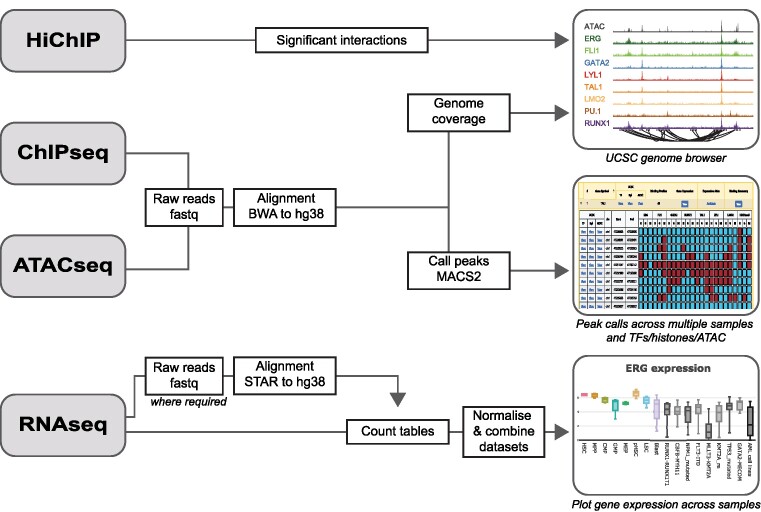
Schematic showing the data analysis pipeline and direct data visualizations included in BloodChIP Xtra.

Data sources are summarized in Supplementary Table S1 (5,17–29), and a schematic showing included datasets is shown in Figure 1. A summary of data analysis and output formats is shown in Figure 2.

### ATACseq data

ATACseq peak coordinates (summit ±250 bp) for healthy human blood populations were downloaded from the gene expression omnibus (GEO) (30) accession GSE74912 (28) and converted from the hg19 to the hg38 reference genome using the UCSC LiftOver tool (31). The resulting 590650 peaks were assigned to genes using the Genomic Regions Enrichment of Annotations Tool (GREAT) v4.0.4 using the basal extension method with default parameters (32,33).

Sequence files downloaded from the Sequence Read Archive (SRA) (34) or ArrayExpress (35) (Supplementary Table S1), were converted to fastq format, and aligned to the GRCh38 genome using BWA v0.7.17 (36). Mitochondrial and duplicate reads were removed using picardMarkDuplicates, reads shifted to account for Tn5-mediated excision using alignmentSieve, replicate samples merged, peaks called with MACS2 v 2.2.7.1 (37) with a minimal threshold *P*-value of 1 × 10^−5^, and tracks for visualization in the UCSC browser generated using deepTools bamCoverage with RPKM normalization (38). Samples from E-MTAB-9021 were single end reads, and in this case, the alignmentSieve step was omitted from the processing pipeline. A synthetic HSC-MPP ATACseq track for visualization on the UCSC browser was generated by merging all bam files from HSC and MPP replicates. ATACseq tracks are available for visualization at http://genome.ucsc.edu/s/PimandaLab/BloodChIP_Xtra_ATAC.

### ChIPseq data

Genome-wide TF and histone binding profiles for acute myeloid leukemia (AML) cell lines KG-1 (22,24), ME-1 (22,25), Kasumi-1 (18–21), TSU-1621-MT (26) were obtained from SRA (Supplementary Table S1). Sequence reads were converted to fastq and processed to remove adapters using cutadapt (39), trimmed to a maximum length of 100 using the command line tool ‘cut’ (reads shorter than 100 were not trimmed), then mapped to the GRCh38 genome using BWA v0.7.17 aligner (36), peaks called using MACS2 v2.2.6 (37), and tracks for visualization in the UCSC browser generated using deepTools’ bamCoverage with RPKM normalization (38). For consistency, all samples were processed as single end sequence data. Genome-wide TF and histone binding profiles for healthy human HSC-MPP, CMP, GMP, MEP were processed as previously described (5). For K27ac and K27me3 peaks were called using the option ‘–broad’. Peak calling controls were composite IgG tracks comprised of either HSC-MPP, CMP, GMP, MEP (for healthy human cells) or KG-1, ME-1 and bulk HSPC (for AML cell lines). ChIPseq tracks are available for visualization at http://genome.ucsc.edu/s/PimandaLab/BloodChIP_Xtra_TF (TFs) and http://genome.ucsc.edu/s/PimandaLab/BloodChIP_Xtra_epigenetics (histones).

### Peak overlaps

Intersecting regions between ChIPseq and ATACseq peaks and the set of 590650 peaks were identified using bedtools intersect (40). An intersection was considered only if >10% of the peak width of the ChIPseq peak overlapped with the ATACseq peak. ATACseq peaks with no corresponding ChIPseq or ATACseq peak in any of the included datasets were then excluded from the final data set which included 387291 peaks.

### Gene expression

Samples with gene expression data available are shown in Supplementary Table S2. RNAseq count tables for healthy human HSPC subpopulations and leukemic subpopulations were downloaded from GEO (GSE74246) (28). RNAseq count tables and corresponding clinical annotation for primary human AML samples (BeatAML2) (41) were downloaded from https://biodev.github.io/BeatAML2/. RNAseq count tables for AML cell lines from the Cancer Cell Line Encyclopedia (42) were downloaded from https://depmap.org/portal/download/all/?releasename=DepMap+Public+23Q2&filename=OmicsExpressionGenesExpectedCountProfile.csv. RNAseq reads for TSU-1621-MT cells (26) were downloaded from SRA (SRR1552863, SRR1552864) and aligned to the human genome (GRCh38.p13; downloaded from https://www.gencodegenes.org/human/release_43.html) using STAR v2.7.9a (43). Gene count tables were normalized using log1p counts per million. Where multiple ENSG ids refer to the same gene symbol, the ENSG id with greater sum total expression over all samples (within that data source) has been reported.

### Database implementation, web interface and availability

The BloodChIP Xtra database is stored and accessed in both Comma-Separated Values (CSV) and Tab-Separated Values (TSV) formats. Our user-friendly web interface, accessible at http://bloodchipXtra.vafaeelab.com/, has been developed using JavaScript, simplifying data access and enabling the visualization of transcription factor (TF) binding and gene expression through Python-based scripts. This interface leverages Plotly (https://plot.ly) for generating downloadable gene expression box plots. The complete dataset is also available for direct download http://bloodchipXtra.vafaeelab.com/downloads/.

## Results: database features

### General web interface

The default BloodChIP Xtra interface displays data filters, search boxes and a table listing all annotated genes in the dataset (Supplementary Figure S1A). The number of annotated ATACseq peaks (Binding Profiles) associated with each gene is shown along with links to the UCSC genome browser to allow visualization of TF, epigenetic (Epi) and ATACseq (ATAC) profiles at that gene locus. The binding status of any combination of TFs/histone and cell types can be displayed simultaneously; the specific binding profiles displayed can be customized using the display filter. A summary table of TF binding at regions associated with each gene or a plot showing expression of individual genes across primary healthy and leukemic cells, and AML-cell lines can also be displayed.

Clicking the arrow expands the gene row to allow exploration of peaks associated with that gene (Supplementary Figure S1B). The genome coordinates of each peak are shown, with red indicating binding of a given TF at that peak in the indicated cell type, and blue indicating no binding peak detected. Peak/cell type combinations with no data are shown in gray.

### Querying the database

The database may be queried by either gene name or TF binding (Supplementary Figure S1A). For gene queries, single genes or multiple genes may be entered. For retrieval of genes potentially regulated by a particular TF in a particular cell type, users can construct a query with any combination of TFs and cell types to identify genes that are combinatorially bound by multiple TFs (‘AND’) or bound by any of the TFs (‘OR’).

### Downstream analysis

To facilitate downstream analysis and data visualization, links are provided to the Expression Atlas (44) to display transcriptomic and proteomic data for the selected gene across multiple datasets. The web interface also allows data export from a queried set of genes; TF binding information can be exported for individual genes, and the normalized expression values of all genes retrieved by a query can be exported to facilitate external analysis such as clustering or generating heatmaps.

### Utility of BloodChIP Xtra: step by step examples

BloodChIP Xtra allows users to query 10 TFs, ATACseq and three histone modifications in eight cell lines. Additional ATACseq profiles for fractionated primary AML cells can be visualized via the UCSC genome browser, and gene expression plots for >800 unique samples spanning normal hematopoiesis and primary and AML cell lines can be displayed for any gene. To start a query, the user first enters the gene/s of interest. For example, searching for the gene, *ERG* returns a single row in the data table, while searching [*ERG GATA2 TAL1*] returns a row for each of those genes (Supplementary Figure S2A). Search hits can then be explored using the Gene Database table.

Rows of the Gene Database table show genes that were returned with the search parameters, and the number of genomic regions which mapped to that gene using GREAT are indicated in the ‘Binding Profiles’ column (Supplementary Figure S2A). Links are provided to pre-compiled sessions in the UCSC browser, and the user may select to visualize TF binding (TF), histone marks, H3K27ac HiChIP, CTCF, RNApolII and STAG2 (Epi) or ATACseq (ATAC) data for all available cell types. Users may also display and visualize gene expression across all cell types by clicking the View button in the Gene Expression column which launches a pop up graph (Supplementary Figure S2B, *ERG* gene). Samples are grouped by cell type, with colours corresponding to the cell types shown in Figure 1. Samples from the BeatAML2 cohort are plotted as a single group, and also as subgroups of clinically relevant mutational subtypes (Supplementary Table S2) (45). Individual cell types can be toggled on and off by clicking on the plot legend. Users may further explore the gene using the link to the Expression Atlas, or view a summary of all binding events assigned to that gene by GREAT (Supplementary Figure S2C, *GATA2* gene).

Additional detail is revealed by clicking the arrow to the left of a gene name which will reveal the binding profiles and cell types selected in the Display Filters check box (Supplementary Figure S2D). The expanded table shows the presence (red) or absence (blue) of a peak for each factor and cell type at all regions mapped to that gene by GREAT (Supplementary Figure S2E). In the example shown for *TAL1* there is pronounced binding of GATA2, RUNX1, TAL1, LYL1 and LMO2 at chr1:47 232 180–47 232 680 which corresponds to the *TAL1* promoter (Supplementary Figure S2F). Grey blocks indicate that no data is available for that factor/cell type combination. Data for any combination of TFs/histones and cell types can be shown simultaneously allowing easy visualization of comparative binding profiles. Additionally, the coordinates of each region are shown and linked to three UCSC browser sessions.

Alternatively, a more complex query can be constructed to return genes bound by selected factors in specific cell types. The ‘Add filter’ button allows multiple factor/cell type combinations to be entered, with AND/OR available to combine queries. Searching, for example, genes bound by the heptad in MEPs retrieves 856 genes; these genes then populate the Gene Database table (Supplementary Figure S2G). For additional analyses the full data tables containing peak calls for 125 factor/cell type combinations and gene expression data for > 800 individual samples are readily available on the download page.

## Discussion

Combinatorial interactions of TFs at enhancers and promoters of co-regulated genes are a core component of the gene regulatory networks which determine cell identity (46). We have recently generated high-quality ChIPmentation and ChIPseq data for ten key TFs and three histone modifications, along with H3K27ac HiChIP, in rare hematopoietic subpopulations spanning HSC commitment along the myeloid/erythroid lineages (5). Here, we have integrated these data with additional TF occupancy, chromatin accessibility and gene expression data from healthy and leukemic cells (Figure 1, Supplementary Table S1) and created a user-friendly database that allows users to (i) query overlapping TF binding at a gene locus of interest, (ii) discover binding coordinates and associated gene targets for TFs of interest and (iii) compare binding profiles between healthy and leukemic cells. Additional features include data downloads and links to further visualize and explore gene expression. The overall aim of this database is to facilitate understanding of dynamic changes to the transcriptional network of HSPCs during lineage commitment along the myeloid/erythroid axis and leukemogenesis.

Understanding the regulatory networks which underlie cell fate decisions, and the ways in which such networks are perturbed in leukemic cells, have important translational implications. For example, bone marrow failure syndromes with underlying genetic causes may be amenable to gene therapy approaches targeting HSCs to produce corrected differentiated cells. However, extensive knowledge of spatiotemporal gene regulation during lineage commitment is needed to avoid lineage skewing due to inappropriate transgene expression. Lineage specific enhancers have been successfully used to overcome this problem and rescue erythroid differentiation in Diamond-Blackfan Anaemia (47). Conversely, gene regulatory networks are perturbed in multiple AML subtypes (1,48), often via recurrent translocation events (49–54), which are increasingly understood to include small structural variants which can mediate enhancer hijacking and oncogenic transformation (55–59). BloodChIP Xtra provides an accessible tool for both discovering lineage specific regulatory elements that may be exploited for gene therapy approaches or understanding the potential clinical consequences of novel structural variants in leukemic cells.

BloodChIP Xtra currently includes healthy HSC-MPP, CMP, GMP and MEP, primary AML and four AML cell lines for which relevant TF binding chromatin and/or accessibility data are available plus gene expression data from >800 healthy and leukemic samples. As additional high-quality datasets are published, the database will be expanded to accommodate additional TF binding profiles and other genome regulators. We believe that BloodChIP Xtra, which integrates combinatorial TF binding with gene expression in normal and malignant cells, will be a useful tool for biologists and bioinformaticians working in hematopoiesis and leukemia and more generally to those working on gene regulation and stem cell biology.

## Supplementary Material

gkad918_Supplemental_Files

## Data Availability

The BloodChIP Xtra database is freely available at http://bloodchipXtra.vafaeelab.com/. The data underlying this article are available in either GEO at https://www.ncbi.nlm.nih.gov/geo/ or ArrayExpress at https://www.ebi.ac.uk/biostudies/arrayexpress. All data accession numbers are provided in Supplementary Table S1.

## References

[1] Thoms J.A.I. , Beck D., Pimanda J.E. Transcriptional networks in acute myeloid leukemia. Genes Chromosomes Cancer. 2019; 58:859–874. 31369171 10.1002/gcc.22794

[2] Pimanda J.E. , Gottgens B. Gene regulatory networks governing haematopoietic stem cell development and identity. Int. J. Dev. Biol. 2010; 54:1201–1211. 20711996 10.1387/ijdb.093038jp

[3] Laurenti E. , Gottgens B. From haematopoietic stem cells to complex differentiation landscapes. Nature. 2018; 553:418–426. 29364285 10.1038/nature25022 PMC6555401

[4] Cornejo-Paramo P. , Roper K., Degnan S.M., Degnan B.M., Wong E.S. Distal regulation, silencers, and a shared combinatorial syntax are hallmarks of animal embryogenesis. Genome Res. 2022; 32:474–487. 35045977 10.1101/gr.275864.121 PMC8896464

[5] Subramanian S. , Thoms J.A.I., Huang Y., Cornejo Paramo C.P., Koch F.C., Jacquelin S., Shen S., Song E., Joshi S., Brownlee C.P. et al. Genome-wide Transcription Factor binding maps reveal cell-specific changes in the regulatory architecture of human HSPC. Blood. 2023; 10.1182/blood.2023021120. PMC10651876 37595278

[6] Doulatov S. , Notta F., Laurenti E., Dick J.E. Hematopoiesis: a human perspective. Cell Stem Cell. 2012; 10:120–136. 22305562 10.1016/j.stem.2012.01.006

[7] Oram S.H. , Thoms J.A., Pridans C., Janes M.E., Kinston S.J., Anand S., Landry J.R., Lock R.B., Jayaraman P.S., Huntly B.J. et al. A previously unrecognized promoter of LMO2 forms part of a transcriptional regulatory circuit mediating LMO2 expression in a subset of T-acute lymphoblastic leukaemia patients. Oncogene. 2010; 29:5796–5808. 20676125 10.1038/onc.2010.320

[8] Curtis D.J. , Salmon J.M., Pimanda J.E. Concise review: blood relatives: formation and regulation of hematopoietic stem cells by the basic helix-loop-helix transcription factors stem cell leukemia and lymphoblastic leukemia-derived sequence 1. Stem Cells. 2012; 30:1053–1058. 22593015 10.1002/stem.1093

[9] Li Y. , Luo H., Liu T., Zacksenhaus E., Ben-David Y. The ets transcription factor Fli-1 in development, cancer and disease. Oncogene. 2015; 34:2022–2031. 24909161 10.1038/onc.2014.162 PMC5028196

[10] Hahn C.N. , Chong C.E., Carmichael C.L., Wilkins E.J., Brautigan P.J., Li X.C., Babic M., Lin M., Carmagnac A., Lee Y.K. et al. Heritable GATA2 mutations associated with familial myelodysplastic syndrome and acute myeloid leukemia. Nat. Genet. 2011; 43:1012–1017. 21892162 10.1038/ng.913 PMC3184204

[11] Pimanda J.E. , Ottersbach K., Knezevic K., Kinston S., Chan W.Y., Wilson N.K., Landry J.R., Wood A.D., Kolb-Kokocinski A., Green A.R. et al. Gata2, Fli1, and Scl form a recursively wired gene-regulatory circuit during early hematopoietic development. Proc. Natl. Acad. Sci. U.S.A. 2007; 104:17692–17697. 17962413 10.1073/pnas.0707045104 PMC2077040

[12] Cai Z. , de Bruijn M., Ma X., Dortland B., Luteijn T., Downing R.J., Dzierzak E. Haploinsufficiency of AML1 affects the temporal and spatial generation of hematopoietic stem cells in the mouse embryo. Immunity. 2000; 13:423–431. 11070161 10.1016/s1074-7613(00)00042-x

[13] Mangan J.K. , Speck N.A. RUNX1 mutations in clonal myeloid disorders: from conventional cytogenetics to next generation sequencing, a story 40 years in the making. Crit. Rev. Oncog. 2011; 16:77–91. 22150309 10.1615/critrevoncog.v16.i1-2.80 PMC3248792

[14] Thoms J.A. , Birger Y., Foster S., Knezevic K., Kirschenbaum Y., Chandrakanthan V., Jonquieres G., Spensberger D., Wong J.W., Oram S.H. et al. ERG promotes T-acute lymphoblastic leukemia and is transcriptionally regulated in leukemic cells by a stem cell enhancer. Blood. 2011; 117:7079–7089. 21536859 10.1182/blood-2010-12-317990

[15] Diffner E. , Beck D., Gudgin E., Thoms J.A., Knezevic K., Pridans C., Foster S., Goode D., Lim W.K., Boelen L. et al. Activity of a heptad of transcription factors is associated with stem cell programs and clinical outcome in acute myeloid leukemia. Blood. 2013; 121:2289–2300. 23327922 10.1182/blood-2012-07-446120

[16] Chacon D. , Beck D., Perera D., Wong J.W., Pimanda J.E. BloodChIP: a database of comparative genome-wide transcription factor binding profiles in human blood cells. Nucleic Acids Res. 2014; 42:D172–D177. 24185696 10.1093/nar/gkt1036 PMC3964976

[17] van der Kouwe E. , Heller G., Czibere A., Pulikkan J.A., Agreiter C., Castilla L.H., Delwel R., Di Ruscio A., Ebralidze A.K., Forte M. et al. Core-binding factor leukemia hijacks the T-cell-prone PU.1 antisense promoter. Blood. 2021; 138:1345–1358. 34010414 10.1182/blood.2020008971 PMC8525333

[18] Mandoli A. , Singh A.A., Prange K.H.M., Tijchon E., Oerlemans M., Dirks R., Ter Huurne M., Wierenga A.T.J., Janssen-Megens E.M., Berentsen K. et al. The hematopoietic transcription factors RUNX1 and ERG prevent AML1-ETO oncogene overexpression and onset of the apoptosis program in t(8;21) AMLs. Cell Rep. 2016; 17:2087–2100. 27851970 10.1016/j.celrep.2016.08.082

[19] Chen Q. , Cevher M.A., Jiang Q., Wang S., Sun X., Roeder R.G., Chen M. LYL1 facilitates AETFC assembly and gene activation by recruiting CARM1 in t(8;21) AML. Proc. Natl. Acad. Sci. U.S.A. 2022; 119:e2213718119. 36215477 10.1073/pnas.2213718119 PMC9586329

[20] Sun X.J. , Wang Z., Wang L., Jiang Y., Kost N., Soong T.D., Chen W.Y., Tang Z., Nakadai T., Elemento O. et al. A stable transcription factor complex nucleated by oligomeric AML1-ETO controls leukaemogenesis. Nature. 2013; 500:93–97. 23812588 10.1038/nature12287 PMC3732535

[21] Ptasinska A. , Assi S.A., Martinez-Soria N., Imperato M.R., Piper J., Cauchy P., Pickin A., James S.R., Hoogenkamp M., Williamson D. et al. Identification of a dynamic core transcriptional network in t(8;21) AML that regulates differentiation block and self-renewal. Cell Rep. 2014; 8:1974–1988. 25242324 10.1016/j.celrep.2014.08.024 PMC4487811

[22] Thoms J.A.I. , Truong P., Subramanian S., Knezevic K., Harvey G., Huang Y., Seneviratne J.A., Carter D.R., Joshi S., Skhinas J. et al. Disruption of a GATA2-TAL1-ERG regulatory circuit promotes erythroid transition in healthy and leukemic stem cells. Blood. 2021; 138:1441–1455. 34075404 10.1182/blood.2020009707

[23] Schaefer E.J. , Wang H.C., Karp H.Q., Meyer C.A., Cejas P., Gearhart M.D., Adelman E.R., Fares I., Apffel A., Lim K. et al. BCOR and BCORL1 Mutations Drive Epigenetic Reprogramming and Oncogenic Signaling by Unlinking PRC1.1 from Target Genes. Blood Cancer Discov. 2022; 3:116–135. 35015684 10.1158/2643-3230.BCD-21-0115 PMC9414116

[24] Minderjahn J. , Schmidt A., Fuchs A., Schill R., Raithel J., Babina M., Schmidl C., Gebhard C., Schmidhofer S., Mendes K. et al. Mechanisms governing the pioneering and redistribution capabilities of the non-classical pioneer PU.1. Nat. Commun. 2020; 11:402. 31964861 10.1038/s41467-019-13960-2 PMC6972792

[25] Mandoli A. , Singh A.A., Jansen P.W., Wierenga A.T., Riahi H., Franci G., Prange K., Saeed S., Vellenga E., Vermeulen M. et al. CBFB-MYH11/RUNX1 together with a compendium of hematopoietic regulators, chromatin modifiers and basal transcription factors occupies self-renewal genes in inv(16) acute myeloid leukemia. Leukemia. 2014; 28:770–778. 24002588 10.1038/leu.2013.257

[26] Sotoca A.M. , Prange K.H., Reijnders B., Mandoli A., Nguyen L.N., Stunnenberg H.G., Martens J.H. The oncofusion protein FUS-ERG targets key hematopoietic regulators and modulates the all-trans retinoic acid signaling pathway in t(16;21) acute myeloid leukemia. Oncogene. 2016; 35:1965–1976. 26148230 10.1038/onc.2015.261 PMC4833872

[27] Beck D. , Thoms J.A., Perera D., Schutte J., Unnikrishnan A., Knezevic K., Kinston S.J., Wilson N.K., O’Brien T.A., Gottgens B. et al. Genome-wide analysis of transcriptional regulators in human HSPCs reveals a densely interconnected network of coding and noncoding genes. Blood. 2013; 122:e12–22. 23974199 10.1182/blood-2013-03-490425

[28] Corces M.R. , Buenrostro J.D., Wu B., Greenside P.G., Chan S.M., Koenig J.L., Snyder M.P., Pritchard J.K., Kundaje A., Greenleaf W.J. et al. Lineage-specific and single-cell chromatin accessibility charts human hematopoiesis and leukemia evolution. Nat. Genet. 2016; 48:1193–1203. 27526324 10.1038/ng.3646 PMC5042844

[29] Pulikkan J.A. , Hegde M., Ahmad H.M., Belaghzal H., Illendula A., Yu J., O’Hagan K., Ou J., Muller-Tidow C., Wolfe S.A. et al. CBFbeta-SMMHC inhibition triggers apoptosis by disrupting MYC chromatin dynamics in acute myeloid leukemia. Cell. 2018; 174:172–186. 29958106 10.1016/j.cell.2018.05.048 PMC6211564

[30] Barrett T. , Wilhite S.E., Ledoux P., Evangelista C., Kim I.F., Tomashevsky M., Marshall K.A., Phillippy K.H., Sherman P.M., Holko M. et al. NCBI GEO: archive for functional genomics data sets–update. Nucleic Acids Res. 2013; 41:D991–995. 23193258 10.1093/nar/gks1193 PMC3531084

[31] Nassar L.R. , Barber G.P., Benet-Pages A., Casper J., Clawson H., Diekhans M., Fischer C., Gonzalez J.N., Hinrichs A.S., Lee B.T. et al. The UCSC Genome Browser database: 2023 update. Nucleic Acids Res. 2023; 51:D1188–D1195. 36420891 10.1093/nar/gkac1072 PMC9825520

[32] McLean C.Y. , Bristor D., Hiller M., Clarke S.L., Schaar B.T., Lowe C.B., Wenger A.M., Bejerano G. GREAT improves functional interpretation of cis-regulatory regions. Nat. Biotechnol. 2010; 28:495–501. 20436461 10.1038/nbt.1630 PMC4840234

[33] Tanigawa Y. , Dyer E.S., Bejerano G. WhichTF is functionally important in your open chromatin data?. PLoS Comput. Biol. 2022; 18:e1010378. 36040971 10.1371/journal.pcbi.1010378 PMC9426921

[34] Katz K. , Shutov O., Lapoint R., Kimelman M., Brister J.R., O'Sullivan C The Sequence Read Archive: a decade more of explosive growth. Nucleic Acids Res. 2022; 50:D387–D390. 34850094 10.1093/nar/gkab1053 PMC8728234

[35] Athar A. , Fullgrabe A., George N., Iqbal H., Huerta L., Ali A., Snow C., Fonseca N.A., Petryszak R., Papatheodorou I. et al. ArrayExpress update - from bulk to single-cell expression data. Nucleic Acids Res. 2019; 47:D711–D715. 30357387 10.1093/nar/gky964 PMC6323929

[36] Li H. , Durbin R. Fast and accurate short read alignment with Burrows-Wheeler transform. Bioinformatics. 2009; 25:1754–1760. 19451168 10.1093/bioinformatics/btp324 PMC2705234

[37] Zhang Y. , Liu T., Meyer C.A., Eeckhoute J., Johnson D.S., Bernstein B.E., Nusbaum C., Myers R.M., Brown M., Li W. et al. Model-based analysis of ChIP-Seq (MACS). Genome Biol. 2008; 9:R137. 18798982 10.1186/gb-2008-9-9-r137 PMC2592715

[38] Ramirez F. , Dundar F., Diehl S., Gruning B.A., Manke T. deepTools: a flexible platform for exploring deep-sequencing data. Nucleic Acids Res. 2014; 42:W187–191. 24799436 10.1093/nar/gku365 PMC4086134

[39] Martin M. Cutadapt removes adapter sequences from high-throughput sequencing reads. EMBnet.journal. 2011; 17:10–12.

[40] Quinlan A.R. , Hall I.M. BEDTools: a flexible suite of utilities for comparing genomic features. Bioinformatics. 2010; 26:841–842. 20110278 10.1093/bioinformatics/btq033 PMC2832824

[41] Bottomly D. , Long N., Schultz A.R., Kurtz S.E., Tognon C.E., Johnson K., Abel M., Agarwal A., Avaylon S., Benton E. et al. Integrative analysis of drug response and clinical outcome in acute myeloid leukemia. Cancer Cell. 2022; 40:850–864. 35868306 10.1016/j.ccell.2022.07.002 PMC9378589

[42] Ghandi M. , Huang F.W., Jane-Valbuena J., Kryukov G.V., Lo C.C., McDonald E.R. 3rd, Barretina J., Gelfand E.T., Bielski C.M., Li H. et al. Next-generation characterization of the Cancer Cell Line Encyclopedia. Nature. 2019; 569:503–508. 31068700 10.1038/s41586-019-1186-3 PMC6697103

[43] Dobin A. , Davis C.A., Schlesinger F., Drenkow J., Zaleski C., Jha S., Batut P., Chaisson M., Gingeras T.R. STAR: ultrafast universal RNA-seq aligner. Bioinformatics. 2013; 29:15–21. 23104886 10.1093/bioinformatics/bts635 PMC3530905

[44] Moreno P. , Fexova S., George N., Manning J.R., Miao Z., Mohammed S., Munoz-Pomer A., Fullgrabe A., Bi Y., Bush N. et al. Expression Atlas update: gene and protein expression in multiple species. Nucleic Acids Res. 2022; 50:D129–D140. 34850121 10.1093/nar/gkab1030 PMC8728300

[45] Dohner H. , Wei A.H., Appelbaum F.R., Craddock C., DiNardo C.D., Dombret H., Ebert B.L., Fenaux P., Godley L.A., Hasserjian R.P. et al. Diagnosis and management of AML in adults: 2022 recommendations from an international expert panel on behalf of the ELN. Blood. 2022; 140:1345–1377. 35797463 10.1182/blood.2022016867

[46] Davidson E.H. Emerging properties of animal gene regulatory networks. Nature. 2010; 468:911–920. 21164479 10.1038/nature09645 PMC3967874

[47] Voit R.A. , Liao X., Cohen B., Armant M., Kamal E., Huang M.-M., Clarke W., Williams D.A., Shimamura A., Sankaran V.G. Regulated Expression of GATA1 As a Gene Therapy Cure for Diamond-Blackfan Anemia. Blood. 2022; 140:986–987.

[48] Assi S.A. , Imperato M.R., Coleman D.J.L., Pickin A., Potluri S., Ptasinska A., Chin P.S., Blair H., Cauchy P., James S.R. et al. Subtype-specific regulatory network rewiring in acute myeloid leukemia. Nat. Genet. 2019; 51:151–162. 30420649 10.1038/s41588-018-0270-1 PMC6330064

[49] Tonks A. , Pearn L., Musson M., Gilkes A., Mills K.I., Burnett A.K., Darley R.L. Transcriptional dysregulation mediated by RUNX1-RUNX1T1 in normal human progenitor cells and in acute myeloid leukaemia. Leukemia. 2007; 21:2495–2505. 17898786 10.1038/sj.leu.2404961

[50] Grinev V.V. , Barneh F., Ilyushonak I.M., Nakjang S., Smink J., van Oort A., Clough R., Seyani M., McNeill H., Reza M. et al. RUNX1/RUNX1T1 mediates alternative splicing and reorganises the transcriptional landscape in leukemia. Nat. Commun. 2021; 12:520. 33483506 10.1038/s41467-020-20848-z PMC7822815

[51] Loke J. , Assi S.A., Imperato M.R., Ptasinska A., Cauchy P., Grabovska Y., Soria N.M., Raghavan M., Delwel H.R., Cockerill P.N. et al. RUNX1-ETO and RUNX1-EVI1 Differentially Reprogram the Chromatin Landscape in t(8;21) and t(3;21) AML. Cell Rep. 2017; 19:1654–1668. 28538183 10.1016/j.celrep.2017.05.005 PMC5457485

[52] Ptasinska A. , Assi S.A., Mannari D., James S.R., Williamson D., Dunne J., Hoogenkamp M., Wu M., Care M., McNeill H. et al. Depletion of RUNX1/ETO in t(8;21) AML cells leads to genome-wide changes in chromatin structure and transcription factor binding. Leukemia. 2012; 26:1829–1841. 22343733 10.1038/leu.2012.49 PMC3419980

[53] Groschel S. , Sanders M.A., Hoogenboezem R., de Wit E., Bouwman B.A.M., Erpelinck C., van der Velden V.H.J., Havermans M., Avellino R., van Lom K. et al. A single oncogenic enhancer rearrangement causes concomitant EVI1 and GATA2 deregulation in leukemia. Cell. 2014; 157:369–381. 24703711 10.1016/j.cell.2014.02.019

[54] Yamazaki H. , Suzuki M., Otsuki A., Shimizu R., Bresnick E.H., Engel J.D., Yamamoto M. A remote GATA2 hematopoietic enhancer drives leukemogenesis in inv(3)(q21;q26) by activating EVI1 expression. Cancer Cell. 2014; 25:415–427. 24703906 10.1016/j.ccr.2014.02.008 PMC4012341

[55] Xu J. , Song F., Lyu H., Kobayashi M., Zhang B., Zhao Z., Hou Y., Wang X., Luan Y., Jia B. et al. Subtype-specific 3D genome alteration in acute myeloid leukaemia. Nature. 2022; 611:387–398. 36289338 10.1038/s41586-022-05365-x PMC10060167

[56] Montefiori L.E. , Bendig S., Gu Z., Chen X., Polonen P., Ma X., Murison A., Zeng A., Garcia-Prat L., Dickerson K. et al. Enhancer hijacking drives oncogenic BCL11B expression in lineage-ambiguous stem cell leukemia. Cancer Discov. 2021; 11:2846–2867. 34103329 10.1158/2159-8290.CD-21-0145 PMC8563395

[57] Ottema S. , Mulet-Lazaro R., Erpelinck-Verschueren C., van Herk S., Havermans M., Arricibita Varea A., Vermeulen M., Beverloo H.B., Groschel S., Haferlach T. et al. The leukemic oncogene EVI1 hijacks a MYC super-enhancer by CTCF-facilitated loops. Nat. Commun. 2021; 12:5679. 34584081 10.1038/s41467-021-25862-3 PMC8479123

[58] Smeenk L. , Ottema S., Mulet-Lazaro R., Ebert A., Havermans M., Varea A.A., Fellner M., Pastoors D., van Herk S., Erpelinck-Verschueren C. et al. Selective requirement of MYB for oncogenic hyperactivation of a translocated enhancer in leukemia. Cancer Discov. 2021; 11:2868–2883. 33980539 10.1158/2159-8290.CD-20-1793 PMC8563373

[59] Botten G.A. , Zhang Y., Dudnyk K., Liu X., Sanders J.T., Imanci A., Droin N., Cao H., Kaphle P., Dickerson K.E. et al. Structural variation cooperates with permissive chromatin to control enhancer hijacking-mediated oncogenic transcription. Blood. 2022; 140:1007–1008. 10.1182/blood.2022017555 PMC10447518 36947815

