# Supplementary material for: BloodChIP Xtra: an expanded database of comparative genome-wide transcription factor binding and gene-expression profiles in healthy human stem/progenitor subsets and leukemic cells
Source: Nucleic Acids Res. 2023 Oct 23;52(D1):D1131–7. doi: 10.1093/nar/gkad918 (PMC10767868; doi:10.1093/nar/gkad918)
Supplement: gkad918_Supplemental_Files [file gkad918_supplemental_files.zip › Thoms et al Supplemental Figures v1.pdf]

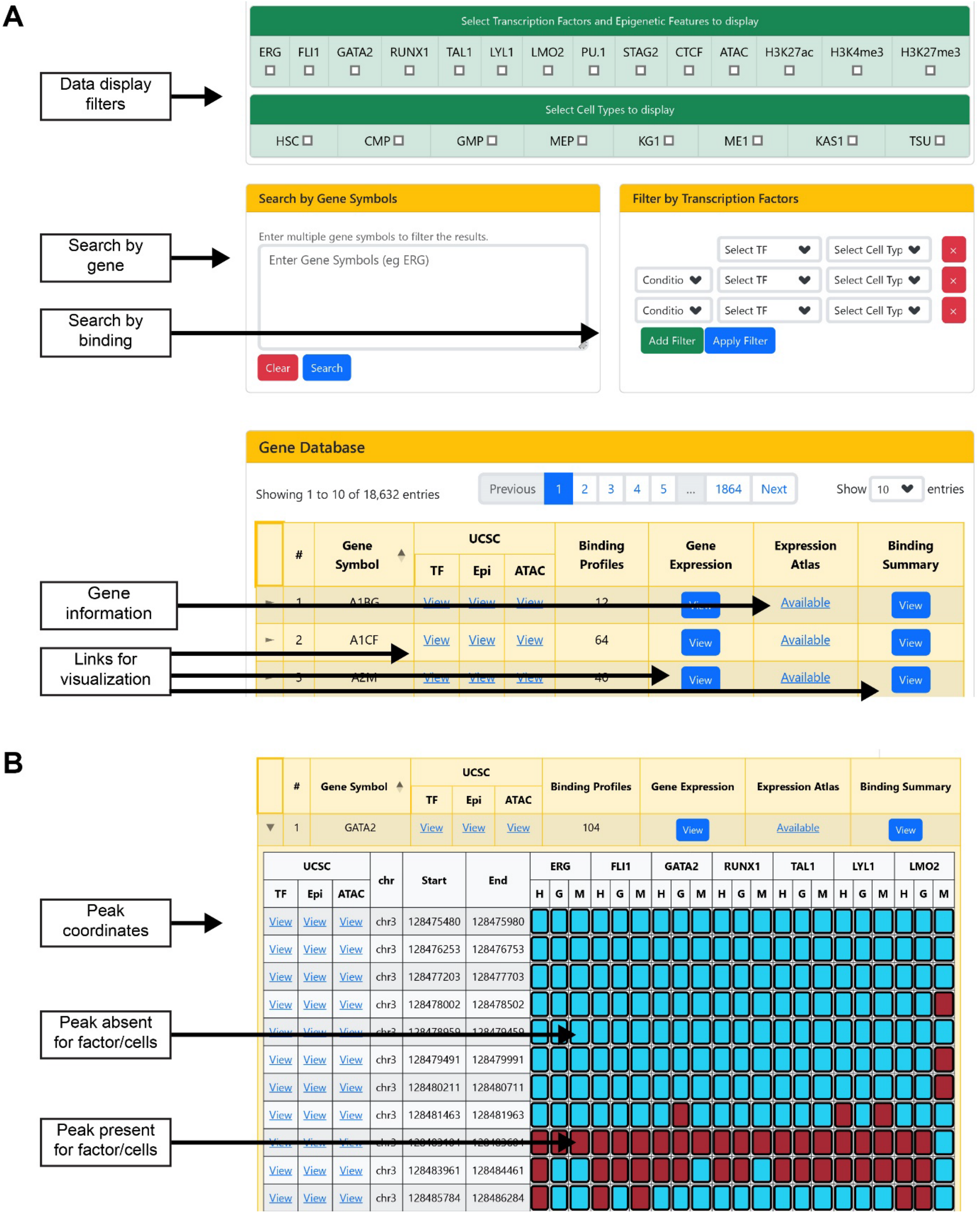

Supplemental Figure 1: Key features of the BloodChIP Xtra web interface. A) Search boxes to search by gene or by TF binding and gene expression. **B)** Comparing TF and histone binding patterns across gene loci and cell types.

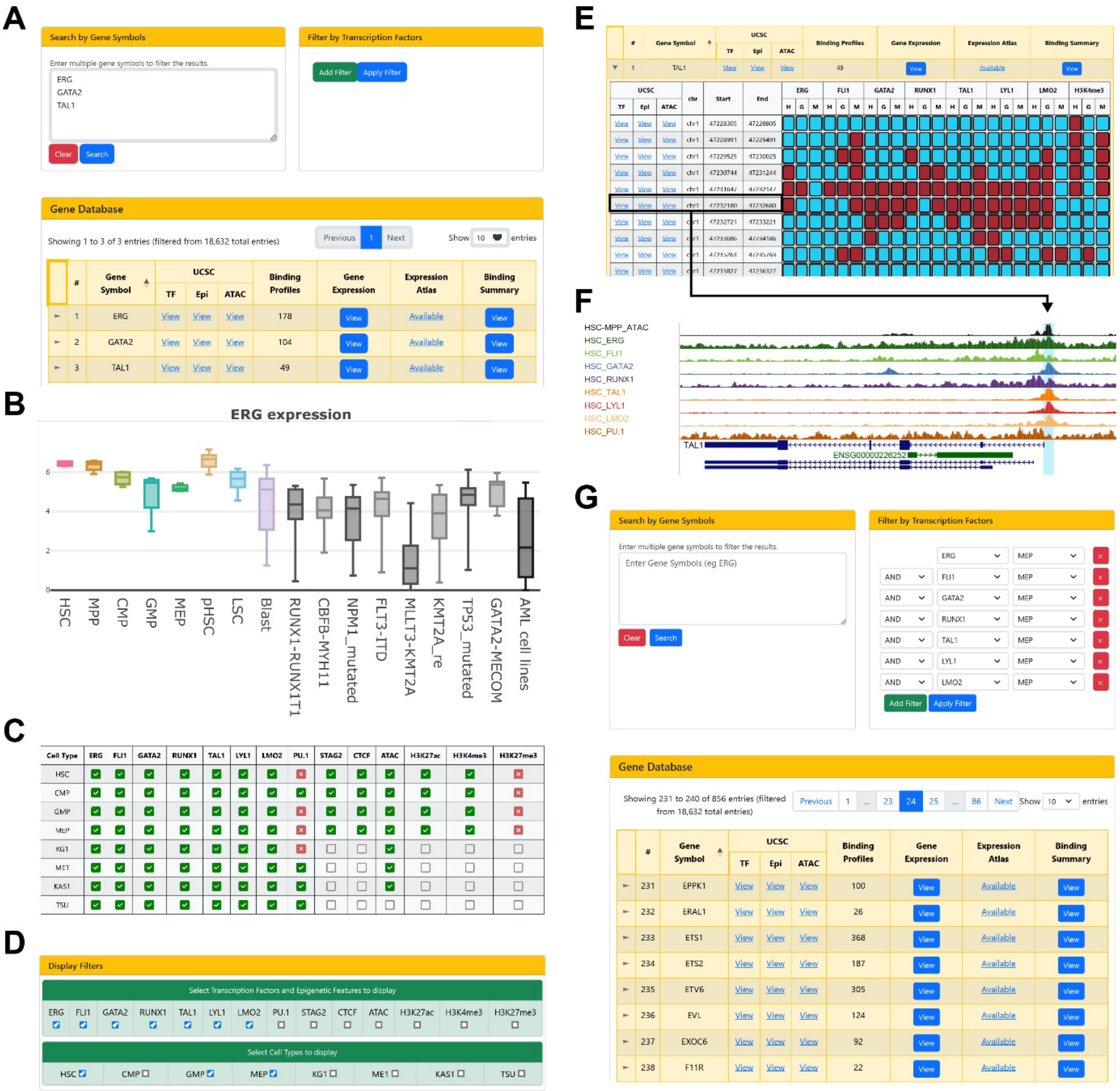

Supplemental Figure 2: Step by step examples of querying BloodChIP Xtra. A) Search by gene name/s. **B)** Cell type specific expression of a selected gene (*ERG*). **C)** Summary of binding events at a selected gene (*GATA2*). **D)** Display filter options. **E)** Binding at regions associated with *TAL1* by GREAT (*not all regions shown*). **F)** UCSC browser showing TF binding at the *TAL1* locus in HSCs. The blue highlighted sections marks chr1:47,232,180-47,232,680, a with combinatorial TF binding in the binding profile summary table (E). **G)** Search for genes heptad-bound genes in MEPs.
